# PairReg: A method for enhancing the learning of molecular structure representation in equivariant graph neural networks

**DOI:** 10.1371/journal.pone.0328501

**Published:** 2025-07-31

**Authors:** Zhen Ren, Yu Liu, Sen Zhang

**Affiliations:** Medical Information Engineering College, Gansu University of Traditional Chinese Medicine, Lanzhou, Gansu, China; Shiv Nadar University, INDIA

## Abstract

The 3D structure of molecules contains a wealth of important information, but traditional 3DCNN-based methods fail to adequately address the transformations of rigid motions (rotation, translation, and mapping). Equivariant graph neural networks (EGNNs) emerge as efficient models to handle molecular 3D structures due to their unique mechanisms for capturing topological properties and equivariance to rigid motions. Historically, the optimization of EGNN models has been achieved by incorporating higher-order features to capture more complex topological properties. However, adding higher-order features incurs high computational costs. To address this issue, we explore the mechanism to mitigate the oversmoothing of equivariant graph neural networks and propose a new method (PairReg) to mitigate oversmoothing by utilizing equivariant information, such as coordinates, to enhance the model’s performance. We validate the performance of the model using the QM9 dataset and conduct ablation experiments on the rMD17 dataset. The results show that our approach enhances the model’s ability to characterize the 3D structure of molecules and offers new insights for enhancing the performance of EGNNs.

## Introduction

Accurate prediction of molecular properties is critical for drug discovery, which can significantly accelerate early drug screening and advance the drug development process. Molecular modeling typically captures the structural features through SMILES [1], 2D molecular graphs [2], and 3D molecular graphs [3, 4]. SMILES strings specify the order and arrangement of connections between atoms, while 2D molecular diagrams are represented as undirected graphs integrating the order of connections between atoms, the type of edges, and a variety of properties. However, SMILES and 2D molecular graphs have limitations in accurately capturing the 3D spatial structure of molecules, and 3D molecular graphs compensate for this by incorporating the spatial coordinates of atoms. Studies [5–7] have shown that fully utilizing the 3D information of molecules is crucial to improving the accuracy of drug design and molecular property prediction.

Graph Neural Networks (GNNs), as powerful machine learning models, have demonstrated excellent performance in a variety of domains such as social network analysis [8, 9], bioinformatics [10, 11], recommender systems [12, 13], and so on. GNNs are able to capture complex relationships among nodes, and have achieved remarkable results in tasks such as node classification, graph classification, and link prediction. Given the wide availability of graph data and the universality of graph structures, GNNs have become a core tool for solving various graph-related tasks.

As GNNs technology advances, Equivariant Graph Neural Networks (EGNNs) [14] have emerged, which are able to fully utilize the 3D spatial information. The EGNNs are equivariant under specific 3D spatial transformations, which enhances their ability to represent 3D structures through equivariant information [15]. Performance degradation of graph neural networks is often attributed to the phenomenon of oversmoothing [16, 17], i.e., as the number of layers increases, the features of the graph nodes converge and fail to capture richer features. Optimization of Equivariant graph neural networks aims to capture richer features by adding higher-order features, but is accompanied by higher computational cost and relatively limited performance improvement. Conversely, there is a gap in the application of oversmoothing mitigation mechanisms to Equivariant graph neural networks. Although some generalized methods exist, these methods often focus on node information and lack the utilization of Equivariant information.

In this study, we propose an effective method to mitigate oversmoothing in Equivariant graph neural networks. By introducing a new regularization technique and a special residual mechanism, our method effectively mitigates the oversmoothing phenomenon while maintaining the Equivariant nature of the model. To validate the effectiveness of our method, we performed experiments for validation on the QM9 dataset and conducted ablation experiments on the rMD17 dataset. The experimental results show that our method improves the performance of Equivariant GNNs, provides examples for mitigating oversmoothing of Equivariant graph neural networks, and offers new perspectives for understanding and improving Equivariant graph neural networks.

### Related work

#### Equivariant graph neural networks.

Molecular 3D structures are typically stored as point clouds, and these point clouds are processed using 3DCNN [18], yet 3DCNN fails to accurately process rotated point clouds. Currently, a leading model for processing molecular 3D structures is the Equivariant Graph Neural Network (EGNN) [14], and incorporating Equivariant information into EGNN can enhance its performance [15]. The Equivariant Graph Neural Network is a special type of graph neural network, which primarily exhibits invariance of scalars and equivariance of vectors. Based on the distinct processing of vector information, Equivariant graph neural networks are typically categorized into two types: Geometric Equivariant Graph Neural Networks and Higher Order Equivariant Graph Neural Networks.

Geometric EGNNs typically extract information based on the distance and angle between two graph nodes. EGNN [14] extracts scalar invariants through the distance between two graph nodes to update node information, while CLofNet [19] and LeftNet [20] map vectors between graph nodes to an invariant local orthogonal coordinate system to obtain scalar invariants for updating node information.

Higher-order Equivariant Graph Neural Networks (EGNNs) are commonly constructed by incorporating higher-order Equivariant features, which serve to enhance the interactions among nodes and to mitigate the loss of information during transmission. The Spherical Harmonic Function (SHF) is utilized by SEGNN [21] to incorporate coordinate information, while the Wigner D-matrix transforms node information into equivariant representations. SEGNN relies solely on Coulomb (CG) coefficients and equivariant information for the update and transmission of node data. MACE [22] extends this approach by generalizing the information exchange from pairwise graph nodes to N-body interactions. TensorNet [23] employs irreducible tensor decomposition based on Cartesian tensors to decompose into rotationally invariant information. Equivariant graph neural networks possess a robust capacity to capture the 3D structure of molecules, yet they are still constrained by the oversmoothing issue inherent in graph neural networks, which prevents them from being applied in deeper layers.Consequently, the number of higher-order Equivariant features and geometric features is limited, and exploring a generalized approach that can enhance Equivariant Graph Neural Networks is both necessary and significant.

#### OverSmooth.

Graph Neural Networks (GNNs) encounter inherent performance issues as their depth increases. The primary manifestation of oversmoothing in GNNs is the reduction of the Dirichlet energy of the graph nodes [16, 17]. Current researchers have primarily addressed the oversmoothing problem through residual connectivity, regularization methods, and early-stopping gating mechanisms.

The residual mechanism has yielded positive outcomes in the deep learning of Euclidean structures, with some researchers [24] exploring ResGCN and DenseGCN. These models exhibit greater stability, albeit with modest performance gains. GCNII [25] enhances the residual mechanism by incorporating the initial value as the residual and introducing unit mapping, thereby enhancing the effect of residuals. Subsequently, some researchers [26, 27] improved the existing residual architecture by incorporating adaptive parameters and a pooling mechanism, achieving some results.

The regularization method primarily involves reducing the Dirichlet energy of each layer through the loss function or potential constraint control, thereby mitigating oversmoothing. PairNorm [28], for instance, subtracts the node feature averages from each layer’s features before passing them to the subsequent layer. Building on this, GraphNormV2 [29] and GRANOLA [30] achieve an adaptive node feature normalization method through the use of trainable normalization parameters. EnergeticGNN [31] confines the Dirichlet energy of each graph layer within specific bounds and introduces a regularization term to penalize the trained weights.

The current optimal performance regarding the oversmoothing problem does not involve a framework that can stably control the Dirichlet energy, G2 [32] proposes a message passing scheme that learns the potential delivery rate, employs a matrix to govern the update of each feature dimension at each node, and uses graph gradient control to ensure convergence to zero as the dimension approaches constancy. Ordered GNN [33] employs both gating and residual mechanisms; for aggregation, it uses chunk gating and a specialized residual mechanism rooted in the node tree. Despite the effectiveness of mechanisms mitigating oversmoothing in graph neural networks, Equivariant graph neural networks possess unexploited equivariant information, and the current approach is limited to invariant information.

## Materials and methods

### FrameWork OverView

**Fig 1:**
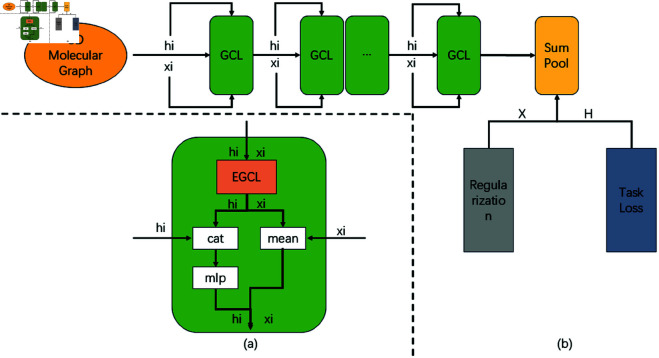
Overall model architecture. Figure (a) presents the detailed diagram of GCL, while Figure (b) illustrates the schematic diagram of the overall framework.

The overall schematic of our model is depicted in Fig 1, where each layer of GCL is connected in a specific manner, and regularization of Equivariant messages is executed at the conclusion. As detailed, the input to the model is the molecular graph, with atom types embedded as node attributes $h_{i}\in {\mathbb{R}}^{f}$ and coordinates $X_{i}\in {\mathbb{R}}^{3}$, and the output is an overarching property of the molecule. EGCL employs a variant of EGNN [14] for fully connected graphs, defining the equations for this layer as follows:

$$m_{ij}=\phi_{e}\left(h_{i}^{l},h_{j}^{l},{\left\|x_{i}^{l}-x_{j}^{l}\right\|}^{2}\right)$$ (1)

$$e_{ij}=\phi_{inf}\left(m_{ij}\right)$$ (2)

$$m_{i}=\sum_{j\in 𝒩(i)}m_{ij}=\sum_{j\neq i}e_{ij}m_{ij}$$ (3)

$$x_{i}^{l+1}=x_{i}^{l}+C\sum_{j\neq i}\left(x_{i}^{l}-x_{j}^{l}\right)\phi_{x}\left(m_{ij}\right)$$ (4)

$$h_{i}^{l+1}=\phi_{h}\left(h_{i}^{l},m_{i}\right)$$ (5)

where $\phi_{inf}$ is a linear layer followed by a sigmoid function. *C* is usually the reciprocal of the number of nodes, averaged over the overall change in coordinates.

### Our method

To address the oversmoothing problem in Equivariant graph neural networks, we first considered traditional residual connections. Based on a series of experiments, we found that the additive residuals, which play a significant role in computer vision, are not applicable to Equivariant graph neural networks, and the concatenation method, which preserves all information, is too computationally expensive, so we ultimately employed a compromise method.

Meanwhile, relative to other graph neural networks, Equivariant graph neural networks possess equivariant information. In geometric Equivariant graph neural networks, the transmission and updating of coordinate information are involved only in specific coordinate regression tasks, while coordinates are frequently neither transmitted nor updated in invariant tasks. We transmit the local deviation by averaging Equivariant information and indirectly regulate the invariant information through the coordinate regression task, which ultimately enhances the model performance. The overall method equation is presented below:

$$h_{i}^{l+1}=\phi_{r}(concat(h_{i}^{l},h_{i}^{l+1}))$$ (6)

$$x_{i}^{l+1}=(x_{i}^{l}+x_{i}^{l+1})/2$$ (7)

$$L_{reg}=\phi_{l}(x^{final},x^{0})$$ (8)

In this context, $\phi_{r}$ transforms the dimension of the merged nodes into that of a single node, and $\phi_{l}$ represents the Euclidean distance used for computing the overall average. Eq 8 was exclusively employed in the final loss calculation. Also, we found that regressing each layer of coordinates to the initial coordinates did not yield satisfactory results, and determined that the regression to the current coordinates was the most stable approach overall.

## Experiments

### QM9

The QM9 dataset [3] is a canonical dataset for predicting chemical properties of molecules within the domain of machine learning, comprising primarily atomic coordinates, atom types (H, C, N, O, F), and molecular properties. The molecular properties are invariant to translations, rotations, and reflections of atomic positions within the molecule, hence most models refrain from updating the coordinates in this dataset.

**Experimental Setup:** We adhere to the dataset preprocessing protocol of EGNN [14], utilizing 100,000 samples for the training set, 18,000 for the validation set, and 12,000 for the testing set. The Adam optimizer is employed with a batch size of 64. The training process spans 1,000 epochs, incorporating learning rate warm-up and cosine annealing. Specifically, the learning rate is warmed up to 1e-4 over the first 250 epochs, followed by cosine annealing to 1e-8 for the remaining epochs.

The primary goal is to evaluate the effectiveness of PairReg in mitigating this issue. To this end, we extend the number of layers in EGNN to nine, which is known to exacerbate oversmoothing. We compare PairReg with two popular oversmoothing mitigation methods: PairNorm [28] and G2 [32]. PairNorm is applied directlyto the node scalar information, while G2 replaces the original graph neural network with EGNN.To more precisely evaluate the relevant performance of our model, we conducted comparisons with SEGNN [21] and ClofNet [19], both of which employ high-order features.

**Results and Analysis:** The results in Table 1 show that our proposed method achieves competitive performance across most attributes compared to SEGNN and ClofNet. Notably, PairReg outperforms both PairNorm and G2 in most cases, demonstrating its effectiveness in mitigating oversmoothing. The experimental results demonstrate the superiority of our proposed method (PairReg) over comparable baseline methods and underscore its significant potential compared to approaches that incorporate higher-order feature interactions. This suggests that leveraging Equivariant information (coordinates) is crucial for addressing oversmoothing in EGNNs.

**Table 1:** Mean absolute error for the molecular property prediction benchmark in QM9 dataset.

Task	α	Δε	$\varepsilon_{HOMO}$	$\varepsilon_{LUMO}$	μ	$C_{\nu }$	*G*	*H*	${\text{R}}^{2}$	*U*	*U*_0_	ZPVE
Units	bohr^3^	meV	meV	meV	D	cal/mol K	meV	meV	bohr^3^	meV	meV	meV
EGNN	.071	48	29	25	.029	.031	12	12	.106	12	11	1.55
SEGNN	.060	42	24	21	.023	.031	15	16	.660	13	15	1.62
ClofNet	.063	53	33	25	.040	.027	9	9	.610	9	8	1.23
EGNN+PairNorm	.088	61	35	31	.032	.037	12	11	.163	11	11	1.86
EGNN+G2	.080	52	30	26	.029	.033	16	15	.149	15	14	1.58
EGNN+PairReg(ours)	.069	49	28	25	.025	.029	10	10	.128	10	9	1.49

### rMD17

The rMD17 dataset [4] is an enhanced iteration of MD17, which diminishes data noise and provides a more accurate depiction of the model’s capabilities. The rMD17 dataset encompasses datasets for ten molecules, with each molecule comprising 100,000 distinct conformations. Each conformation is characterized by atomic coordinates, atom types, molecular energy, and force field data.

**Experimental Setup:** For the two experiments conducted on this dataset, we employ an identical preprocessing and training strategy. Molecular graphs are constructed using one-hot encodings of atoms, atomic numbers, and atomic coordinates within the molecules. The dataset is partitioned into 80,000 samples for the training set, 10,000 for the validation set, and 10,000 for the testing set.The Adam optimizer is utilized with a batch size of 96. The training process spans 1,000 epochs, incorporating learning rate warm-up and cosine annealing. Specifically, the learning rate is warmed up to 1e-4 over the first 250 epochs, followed by cosine annealing to 1e-8 for the remaining epochs. For the ablation experiments, we continue to employ a 9-layer EGNN.

We conduct two experiments on the rMD17 dataset. First, we investigate the performance degradation of EGNN as the number of layers increases. Second, we evaluate the effectiveness of PairReg in mitigating this degradation. We also perform extensive ablation experiments to validate PairReg by separately analyzing the processing of scalar and Equivariant information.

**Fig 2:**
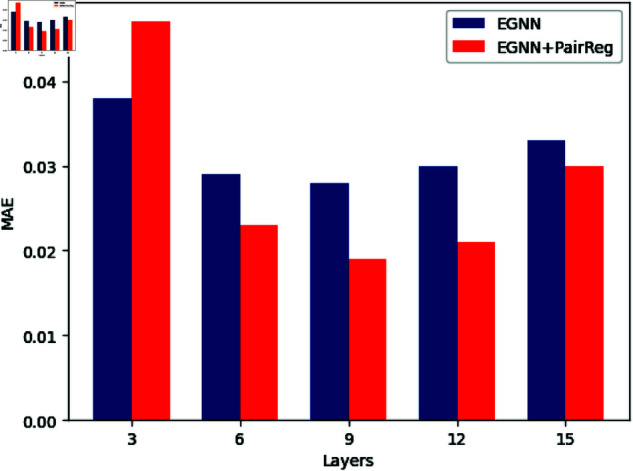
Model predictions in rMD17-malonaldehyde.

**Results and Analysis:** As shown in Fig 2, EGNN exhibits significant performance degradation as the number of layers increases due to oversmoothing. However, our proposed PairReg method effectively alleviates this issue, improving model performance across all tested layers. This demonstrates PairReg’s ability to mitigate oversmoothing in deep EGNNs.

The ablation experiments in Table 2 further validate PairReg’s effectiveness. When only node features are used (Node Only), the model’s performance degrades significantly, likely due to oversmoothing exacerbated by residual concatenation. In contrast, combining node features and coordinates (Full) yields the best performance, highlighting the importance of leveraging both scalar and Equivariant information. These results confirm that PairReg effectively balances these two types of information, leading to improved model performance in deep networks.

**Table 2:** Mean absolute error for the molecular Energy (meV) prediction benchmark in rMD17 dataset.

Molecule	Full	Coord Only	Node Only
Aspirin	2.51	3.55	5.24
Azobenzene	1.30	1.95	2.99
Benzene	0.30	0.52	0.82
Ethanol	0.39	0.60	1.08
Malonaldehyde	0.82	1.12	2.08
Naphthalene	0.95	1.77	2.16
Paracetamol	1.51	3.03	3.46
Salicylic acid	0.91	1.17	2.34
Toluene	0.52	0.82	1.47
Uracil	0.60	0.95	1.51

## Conclusion

Incorporating higher-order features can enhance the performance of Equivariant Graph Neural Networks (EGNNs), yet this may escalate computational costs. Our study concentrates on alleviating the over-smoothing phenomenon in EGNNs at a minimal computational expense to bolster the precision of molecular attribute prediction. Empirical evidence from the QM9 dataset indicates that our approach achieves a substantial improvement in the majority of molecular attribute prediction tasks. The rMD17 dataset results demonstrate that our method effectively counters over-smoothing and augments the model’s predictive capabilities for molecular attributes, particularly when performance deterioration is observed. Our research successfully establishes that the efficacy of EGNNs in molecular property prediction can be significantly enhanced by addressing the over-smoothing issue, a discovery that not only substantiates the viability of our approach but also paves the way for future investigations.

In the future, we intend to broaden the model’s application to biomolecule binding strength prediction and drug molecule generation, potentially hastening the drug discovery process. The characteristics of biomacromolecules impose novel challenges on our molecular prediction model, underscoring the need to investigate its application in the context of large-scale graph data. Given that EGNNs leveraging higher-order features often omit the transfer of coordinate information, the integration of our approach within such networks warrants further exploration.

## References

[1] Weininger D. SMILES, a chemical language and information system. 1. Introduction to methodology and encoding rules. J Chem Inf Comput Sci. 1988;28(1):31–6.

[2] Wu Z, Ramsundar B, Feinberg EN, Gomes J, Geniesse C, Pappu AS, et al. MoleculeNet: a benchmark for molecular machine learning. Chem Sci. 2017;9(2):513–30. doi: 10.1039/c7sc02664a 29629118 PMC5868307

[3] Ramakrishnan R, Dral PO, Rupp M, Von Lilienfeld OA. Quantum chemistry structures and properties of 134 kilo molecules. Scientific Data. 2014;1(1):1–7. 10.1038/sdata.2014.22 PMC4322582 25977779

[4] Christensen AS, Von Lilienfeld OA. On the role of gradients for machine learning of molecular energies and forces. Mach Learn: Sci Technol. 2020;1(4):045018.

[5] Li Y, Pei J, Lai L. Synthesis-driven design of 3D molecules for structure-based drug discovery using geometric transformers. arXiv preprint 2022. doi: arXiv:230100167

[6] Song T, Ren Y, Wang S, Han P, Wang L, Li X, et al. DNMG: deep molecular generative model by fusion of 3D information for de novo drug design. Methods. 2023;211:10–22. doi: 10.1016/j.ymeth.2023.02.001 36764588

[7] Kuang T, Ren Y, Ren Z. 3D-Mol: a novel contrastive learning framework for molecular property prediction with 3D information. Pattern Anal Applic. 2024;27(3). doi: 10.1007/s10044-024-01287-8

[8] Fan W, Ma Y, Li Q, He Y, Zhao E, Tang J, et al. Graph neural networks for social recommendation. In: The World Wide Web Conference. 2019. p. 417–26. 10.1145/3308558.3313488

[9] Fan W, Ma Y, Li Q, Wang J, Cai G, Tang J, et al. A graph neural network framework for social recommendations. IEEE Trans Knowl Data Eng. 2022;34(5):2033–47. doi: 10.1109/tkde.2020.3008732

[10] Zhang X-M, Liang L, Liu L, Tang M-J. Graph neural networks and their current applications in bioinformatics. Front Genet. 2021;12:690049. doi: 10.3389/fgene.2021.690049 34394185 PMC8360394

[11] Yi H-C, You Z-H, Huang D-S, Kwoh CK. Graph representation learning in bioinformatics: trends, methods and applications. Brief Bioinform. 2022;23(1):bbab340. doi: 10.1093/bib/bbab340 34471921

[12] Wu S, Sun F, Zhang W, Xie X, Cui B. Graph neural networks in recommender systems: a survey. ACM Comput Surv. 2022;55(5):1–37. doi: 10.1145/3535101

[13] Gao C, Zheng Y, Li N, Li Y, Qin Y, Piao J, et al. A survey of graph neural networks for recommender systems: challenges, methods, and directions. ACM Trans Recomm Syst. 2023;1(1):1–51. doi: 10.1145/3568022

[14] Satorras VG, Hoogeboom E, Welling M. E (n) equivariant graph neural networks. In: International conference on machine learning. PMLR; 2021. p. 9323–32.

[15] Joshi CK, Bodnar C, Mathis SV, Cohen T, Lio P. On the expressive power of geometric graph neural networks. In: International conference on machine learning. PMLR; 2023. p. 15330–55.

[16] Chen D, Lin Y, Li W, Li P, Zhou J, Sun X. Measuring and relieving the over-smoothing problem for graph neural networks from the topological view. AAAI. 2020;34(04):3438–45. doi: 10.1609/aaai.v34i04.5747

[17] Rusch TK, Bronstein MM, Mishra S. A survey on oversmoothing in graph neural networks. arXiv preprint 2023. doi: arXiv:230310993

[18] Guo Y, Wang H, Hu Q, Liu H, Liu L, Bennamoun M. Deep learning for 3D point clouds: a survey. IEEE Trans Pattern Anal Mach Intell. 2021;43(12):4338–64. doi: 10.1109/TPAMI.2020.3005434 32750799

[19] Du W, Zhang H, Du Y, Meng Q, Chen W, Zheng N. SE (3) equivariant graph neural networks with complete local frames. In: International Conference on Machine Learning. PMLR; 2022. p. 5583–608.

[20] Du Y, Wang L, Feng D, Wang G, Ji S, Gomes CP. A new perspective on building efficient and expressive 3D equivariant graph neural networks. Adv Neural Inf Process Syst. 2024;36.

[21] Brandstetter J, Hesselink R, van der Pol E, Bekkers EJ, Welling M. Geometric and physical quantities improve e (3) equivariant message passing. arXiv preprint 2021. https://arxiv.org/abs/2110.02905

[22] Batatia I, Kovacs DP, Simm G, Ortner C, Csányi G. MACE: higher order equivariant message passing neural networks for fast and accurate force fields. Adv Neural Inf Process Syst. 2022;35:11423–36.

[23] Simeon G, De Fabritiis G. Tensornet: cartesian tensor representations for efficient learning of molecular potentials. Adv Neural Inf Process Syst. 2024;36.

[24] Li G, Muller M, Thabet A, Ghanem B. Deepgcns: can gcns go as deep as cnns? In: Proceedings of the IEEE/CVF International Conference on Computer Vision; 2019. p. 9267–76.

[25] Chen M, Wei Z, Huang Z, Ding B, Li Y. Simple and deep graph convolutional networks. In: International conference on machine learning. PMLR; 2020. p. 1725–35.

[26] Liu X, Ding J, Jin W, Xu H, Ma Y, Liu Z. Graph neural networks with adaptive residual. Adv Neural Inf Process Syst. 2021;34:9720–33.

[27] Duan Y, Wang J, Ma H, Sun Y. Residual convolutional graph neural network with subgraph attention pooling. Tsinghua Sci Technol. 2021;27(4):653–63.

[28] Zhao L, Akoglu L. Pairnorm: tackling oversmoothing in GNNs. arXiv preprint 2019. https://arxiv.org/abs/1909.12223

[29] Scholkemper M, Wu X, Jadbabaie A, Schaub M. Residual connections and normalization can provably prevent oversmoothing in GNNs. arXiv preprint 2024. https://arxiv.org/abs/240602997

[30] Eliasof M, Bevilacqua B, Schönlieb CB, Maron H. GRANOLA: adaptive normalization for graph neural networks. arXiv preprint 2024. https://arxiv.org/abs/2404.13344

[31] Zhou K, Huang X, Zha D, Chen R, Li L, Choi SH. Dirichlet energy constrained learning for deep graph neural networks. Adv Neural Inf Process Syst. 2021;34:21834–46.

[32] Rusch TK, Chamberlain BP, Mahoney MW, Bronstein MM, Mishra S. Gradient gating for deep multi-rate learning on graphs. arXiv preprint 2022. https://arxiv.org/abs/2210.00513

[33] Song Y, Zhou C, Wang X, Lin Z. Ordered gnn: ordering message passing to deal with heterophily and over-smoothing. arXiv preprint 2023. https://arxiv.org/abs/2302.01524

